# EGLN1-positive familial erythrocytosis: a rare variant with an unusually aggressive clinical course

**DOI:** 10.1007/s12308-025-00645-7

**Published:** 2025-07-08

**Authors:** Laura Maule, Brielle Coe, Rishi Sawhney

**Affiliations:** 1 Drexel University, Philadelphia, United States; 2 Bayhealth Medical Center, Dover, United States

**Keywords:** Familial erythrocytosis type 3, Germline mutations, PHD2

## Abstract

Familial erythrocytosis type 3 (ECYT3) is a rare condition caused by loss of function germline mutations in the prolyl hydroxylase domain-2 (PHD2), a regulator in the hypoxia-sensing pathway. Although mutations in PHD2 have been previously described, this particular variant lacks clinical characterization and presents with an aggressive course. We report the case of a patient with vasomotor symptoms and elevations in hematocrit (HCT) and hemoglobin (Hgb) despite frequent therapeutic phlebotomy. He had a family history of erythrocytosis spanning four generations. Germline genetic testing revealed a rare pathogenic variant of PHD2, confirming a diagnosis of ECYT3. Therapeutic phlebotomy yielded only transient Hgb and HCT reductions and only partial symptomatic control. This case highlights the diagnostic challenges and limitations of current treatments for hereditary erythrocytosis and underscores the need for symptom-centered management strategies. Furthermore, we highlight a gap in the literature around the pathophysiology and management of ECYT3.

## Introduction

The hypoxia-sensing pathway is an important regulator of erythrocytosis. In hypoxic conditions, hypoxia-inducible factor (HIF) acts as a transcription factor, binding to the enhancer region of erythropoietin (EPO), stimulating red blood cell (RBC) production to increase oxygen delivery to tissues. When oxygen levels are normal, PHD2 hydroxylates HIF, attracting VHL (von-Hippen Lindau) to bind. VHL ubiquitinates HIF for proteasomal destruction. The net effect is halted erythropoiesis. Pathogenic variants of the components of this pathway can create a pseudohypoxic state, leading to aberrant erythropoiesis [1–5]. ECYT3 has been designated as a distinct disease by the Online Mendelian Inheritance in Man (OMIM) [MIM #609820] associated with erythrocytosis, thrombosis, and vasomotor symptoms. It is caused by autosomal dominant germline mutations in PHD2, encoded by the egl-9 family hypoxia inducible factor 1 (EGLN1) gene [5, 6]. PHD2 underactivity precludes HIF from ubiquitination by VHL, leading to erythrocytosis.

Due to its rarity and limited availability of germline genetic testing, information on ECYT3 in the literature is limited. Several variants of PHD2 have been described, and knowledge of the clinical patterns in each variant is lacking. This case report describes a case of a patient with a strong family history of erythrocytosis, who was diagnosed with a rare variant of ECYT3. We provide the first detailed clinical description of this germline mutation and describe our treatment and outcomes.

## Clinical history

The patient presented to Bayhealth Medical Center in Dover, DE, with a known diagnosis of polycythemia. He reported a chronic and debilitating constellation of symptoms, including persistent fatigue, recurrent headaches, aquagenic pruritus, and periodic abdominal discomfort. These symptoms persisted despite therapeutic phlebotomies performed every 4 to 6 weeks. He experienced only partial and temporary relief when his hematocrit was reduced to a target of 38–40%, below which he developed symptoms of anemia including dizziness and near-syncope. Even with this aggressive management, his symptoms continued to worsen between treatments and did not respond to conventional symptomatic therapies such as cimetidine and hydroxyzine.

His past medical history included hypercholesterolemia, gastroesophageal reflux disease, and obstructive sleep apnea, which was well controlled with consistent use of CPAP. He also had a history of orthopedic surgeries, including rotator cuff and anterior cruciate ligament repairs. The patient was a lifelong non-smoker and had no history of alcohol use disorder. He denied any occupational exposure to toxins but did report substantial secondhand smoke exposure throughout his childhood. His family history was notable for erythrocytosis affecting four generations, including his maternal grandfather, mother, and son. Genetic testing eventually confirmed that his son was positive for an *EGLN1* gene variant associated with familial erythrocytosis.

On physical examination, the only notable finding was left upper quadrant tenderness but no palpable hepatosplenomegaly. Laboratory evaluation showed a hematocrit of 51.2 percent, hemoglobin of 17.8 g per deciliter, RBC count of 5.82 million per microliter, white blood cell count of 6.3 thousand per microliter, platelet count of 142,000 per microliter, and mean corpuscular volume of 88.1 femtoliters. His erythropoietin level was 7.3 milli-international units per milliliter. Review of the peripheral blood smear showed normal morphology of red blood cells, white blood cells, and platelets. Historical laboratory data consistently demonstrated hemoglobin and hematocrit levels in the high-normal range.

## Materials and methods

**Fig. 1:**
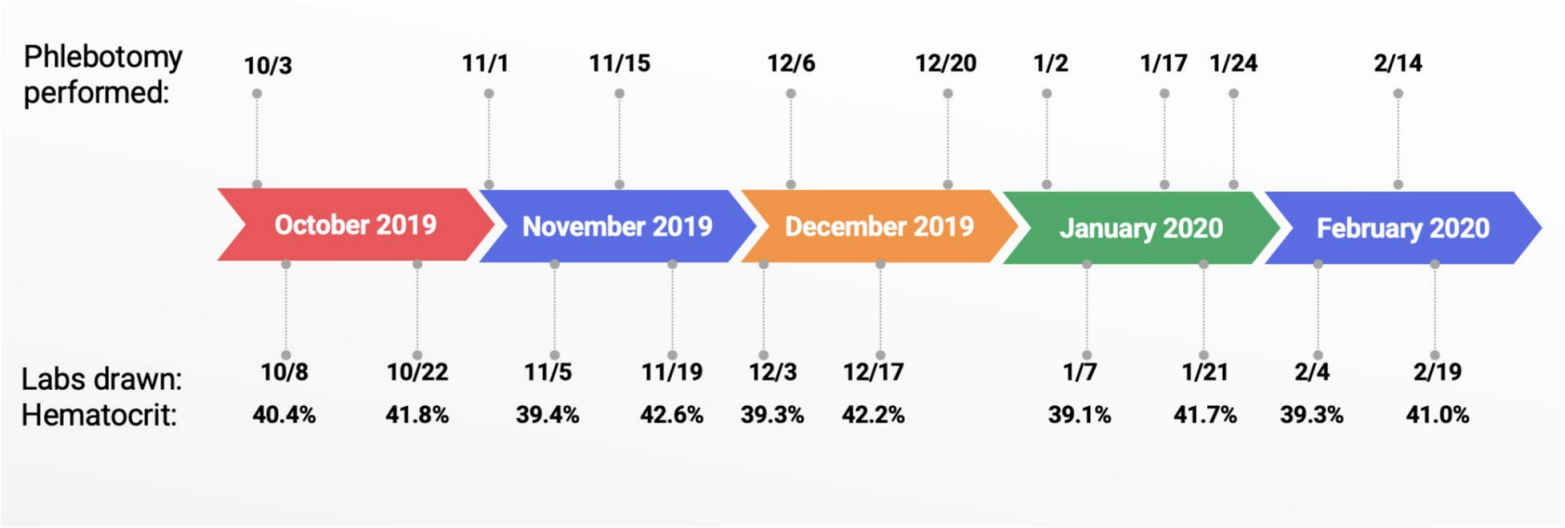
The patient’s frequent phlebotomy illustrated from October 2019 to February 2020 with 9 venesections performed during this time

Clinical details were manually extracted from electronic health records. Some labs were sent out outside laboratories. Bone marrow biopsies were taken as core needle biopsies and stained with H&E, myeloperoxidase, reticulin, CD20, CD3, CD34, and CD61 antibodies. Giemsa smears were prepared to evaluate hematopoietic precursors. Five FISH assays were completed to identify chromosomal abnormalities. A three-color probe that hybridizes to BCR, ABL1, and ASS1 was used on interphase nuclei to identify BCR/ABL1 rearrangements. Additional probes for MYC, CDKN2A, PTPRT deletions, and D13S319 deletions were used to identify other mutations commonly found in myelodysplastic syndromes, myeloproliferative neoplasms, and acute myeloid leukemia. Flow cytometry on bone marrow aspirate measured relative quantities of CD4 + and CD8 + T cells, B cells, monocytes (CD14, CD64), granulocytes (CD56, CD57), plasma cells (CD38), and hematopoietic precursors (CD34, CD117). Lymphocytes and maturing myeloid cells were gated using CD45-APC-H7 staining intensity and side scatter (Fig. 1). Genomic DNA was isolated and polymerase chain reaction (PCR) with Sanger sequencing was used to analyze whole peripheral blood samples for JAK2 exon 12, exon 13, and V617F mutations. PCR with Sanger sequencing was also used to analyze the patient’s genomic DNA for W515 and S505 mutations in MPL and CALR, BPGM, EGLN1, EPOR gene, EPAS1 (HIF2A gene), and VHL.

## Results

Initial molecular testing for common mutations including JAK2 (V617F, exon 12, exon 13 mutations) yielded normal results, reducing the likelihood of polycythemia vera and other acquired myeloproliferative disorders, though rare variants cannot be excluded. Two bone marrow biopsies confirmed normal cellularity with trilineage hematopoiesis with no evidence of fibrosis and negative molecular studies including JAK2, CALR, MPL, and BCR/ABL. Karyotyping demonstrated normal 46,XY karyotype. Testing for monoclonal gammopathies indicative of TEMPI (telangiectasias, erythrocytosis with elevated EPO, monoclonal gammopathy, perinephric fluid collection, and intrapulmonary shunting) syndrome was negative.

Despite comprehensive evaluation of uncontrolled causes of secondary erythrocytosis, the patient’s symptoms remained unexplained, suggesting the possibility of a hereditary erythrocytosis. Germline genetic testing for rare congenital erythrocytosis disorders revealed a rare mutation, c.494del p(Pro165Glnfs*9), a frameshift mutation in exon 1 of EGLN1, consistent with ECYT3. Other variants of ECYT3 follow an autosomal dominant inheritance pattern, suggesting a common etiology in this patient’s strong family history of erythrocytosis [5, 6].

## Discussion

The c.494del p(Pro165Glnfs*9) mutation has been reported in only two prior studies, but the phenotype remains poorly characterized [2, 7]. Oliviera et al. described one patient with an identical variant who presented with splenomegaly, portal vein thrombosis, headache, Hgb 17.1 g/dL, and EPO 51 mlU/mL [2]. However, clinical courses, management, and outcomes are unknown. In Dewey et al., of 22 individuals with unspecified frameshift mutations in Pro165 of EGLN1, only seven patients had erythrocytosis, and there is no further discussion. Mutations in PHD2 have also been implicated in cases of paragangliomas indicating tumor suppressor activity, but it remains unclear whether patients with ECYT3 have an increased risk for these tumors [8, 9].

Although this patient’s presentation is largely similar to other variants of ECYT3, several key features set this case apart. The patient’s severe and refractory vasomotor symptom burden was out of proportion to the aggressive hematocrit target of 38–40%. Symptom control required a delicate balance between avoiding erythrocytosis symptoms and inducing anemia. Some symptoms, namely pruritus and headache, could not be entirely controlled by routine venesection and have been unresponsive to alternative medications.

His medical history has since been complicated by multiple thrombotic events, raising concerns for an underlying hypercoagulable state. He experienced a retinal artery occlusion, venous insufficiency, and thrombophlebitis following superficial vein thrombosis. Taken together, these incidents combined with the patient’s persistent erythrocytosis suggest a predisposition to thrombophilia in ECYT3.

This patient’s EPO was normal at the time of testing, a counterintuitive finding given PHD2’s role in suppressing EPO production. Similar findings were reported in other cases of ECYT3, though none with this particular variant [10]. The only other documented case of this variant with laboratory data had an abnormally high erythropoietin [2]. One hypothesis is that erythrocytosis with low to normal EPO could point towards increased sensitivity or expression of the EPO receptor [10]. A second theory is that ECYT3 creates a new HCT “set-point,” causing an elevation of EPO after phlebotomy, returning to normal once the patient reaches that set point. This phenomenon was observed in a subset of patients with familial erythrocytosis type 2 (ECYT2), defined by loss of function of VHL [3, 11, 12].

This case study illustrates the complexity of ECYT3 through a comprehensive patient journey spanning nearly two decades. Our patient has provided a detailed family and personal medical history and documentation of his symptoms. However, there are several limitations, including a lack of familial data. Anecdotally, his son had the same variant of PHD2 with similar symptoms, though his health information is unknown. More clinical data and further studies of patients with ECYT3 are necessary to understand the pathophysiology and ideal treatment, particularly with the c.494del p(Pro165Glnfs*9) variant. To improve our understanding of ECTY3 and identify appropriate treatments, we urge providers to test patients with unexplained erythrocytosis for germline genetic mutations. Clinical findings in patients harboring germline variants in PHD2 should be reported to build a stronger evidence base and inform future studies on ECYT3.

## Data Availability

No datasets were generated or analyzed during the current study.
